# Association between *ACE *gene I/D polymorphisms and hyperandrogenism in women with Polycystic Ovary Syndrome (PCOS) and controls

**DOI:** 10.1186/1471-2350-10-64

**Published:** 2009-07-14

**Authors:** Jing sun, Haijian fan, Yena Che, Yunxia Cao, Xiaoke Wu, Hai-xiang Sun, Fengjing Liang, Long Yi, Yong Wang

**Affiliations:** 1 Jiangsu Key Laboratory of Molecular Medicine & The reproductive medicine Center of Drum Tower Hospital, Medical School of Nanjing University, Nanjing 210093, PR China; 2 Department of Obstetrics and Gynecology, Anhui Medical University, Hefei 230022, PR China; 3 Department of Obstetrics and Gynecology, The First Affiliated Hospital, Heilongjiang University of Chinese Medicine, Harbin 150040, PR China

## Abstract

**Background:**

I/D polymorphisms of *ACE *are associated with the plasma ACE concentration. The ACE is associated with the angiogenesis of ovarian endothelium in vitro as well as steroidogenesis and follicular growth in cattle. Since ACE induces a high blood supply and hypersteroidogenesis in the ovary, it may be associated with polycystic ovary syndrome (PCOS) which exhibits hyperplasia, hypervascularity of the ovarian theca interna and stroma, as well as disorderd steroidogenesis. Therefore, we hypothesized that the ACE plays some roles in the human ovary. To investigate whether the *ACE *I/D polymorphisms are associated with the steroidogenesis disorder in PCOS and contribute to the susceptibility of PCOS in Chinese women, we designed a case-controlled association study in 582 individuals.

**Methods:**

The *ACE *I/D polymorphisms were assessed in 582 reproductive-age women. Genotyping and frequency of *ACE *I/D polymorphisms were obtained by PCR amplification that was performed on genomic DNA isolated from blood leucocytes. Results were analyzed in respect to clinical test results.

**Results:**

The frequencies of the D allele and the genotypic distributions (DD, ID and II) in the women with PCOS did not differ from those in controls (*P *= 0.458). However, there were significant differences in the concentrations of testosterone among three genotypes both in the PCOS patients and controls (*P *= 0.0045, *P *= 0.0052, respectively). Differences were also found between these groups with distinct genotypes: DD versus II and DI versus II in the PCOS patients as well as DD versus DI and DD versus II in the controls. There were significant differences in the ratio of LH/FSH among three genotypes in the patients (*P *= 0.01). However, there were no statistical differences in the BMI, AAM, E2 concentrations and other serum hormone concentrations among the three genotypes both in the PCOS patients and controls.

**Conclusion:**

The *ACE *I/D polymorphisms were not associated with the pathogenesis of PCOS. However, the polymorphisms were associated with the steroidogenesis in the ovary. The observation indicated that the *ACE *I/D polymorphisms were not the key etiological factor, which in stead may be associated with the aggravated clinical manifestations of PCOS.

## Background

There is evidence to indicate that the RAS may influence oocyte maturation, ovulation and steroidogenesis as well as formation of corpus luteum through complex interactions with other systems [1]. ACE, encoded by the *ACE *gene, is one of the components of RAS and can be expressed in multiple tissues including ovaries [2]. In addition to the important role in regulating blood pressure, the ACE and its products are associated with the angiogenesis of ovarian endothelium in vitro [3] and the resumption of meiosis [4,5], steroidogenesis [6], and follicular growth [7] in cattle. However, there are few reports on the roles of ACE in human ovaries. Rigat et al [8] demonstrated that the inter-individual variability of the plasma ACE concentration is associated with an insertion (I)/deletion (D) polymorphism involving a 287-bp DNA sequence situated in intron 16 of the *ACE *gene, the so-called *ACE *I/D polymorphism. Thus researchers use the I/D polymorphism as a valid marker for studying the associations between the *ACE *gene polymorphism(s) and pathophysiological conditions.

PCOS, present in 5%–10% of reproductive-age women, is a constellation of hyperandrogenism, hyperinsulinism [9,10], menstrual dysfunction, and associated metabolic and cardiovascular complications [11]. It is postulated that the environmental and genetic factors contribute to the etiology of PCOS [12]. During the past decades, the roles of more than 70 candidate genes have been evaluated for causal roles in PCOS; however, because of genetic and phenotypic heterogeneity and underpowered studies, the results of many of these studies remain inconclusive.

Patients with PCOS not only have the clinical characteristics of oligomenorrhoea, hirsutism, infertility and hyperinsulinemia but also exhibit obesity, hypertension, dyslipidemia, and an increased pro-thrombotic state [10,11]. They also have an increased risk of type2 diabetes [13], impaired glucose tolerance and cardiovascular diseases. Hyperplasia and hypervascularity of the ovarian theca interna and stroma are also the prominent features of PCOS, a leading cause of infertility [14]. As an important regulator of blood pressure, ACE may play a potential role in the progress of the cardiovascular diseases in the patients with PCOS. In addition, the product of the ACE- Angiotensin II plays a role in angiogenesis during ovulation, the formation of the corpus luteum, and luteolysis in cattle [15]. Early studies using pharmacologic approaches indicated that receptors for angiotensin II are present on steroidogenic cells which can synthesize steroid hormones [1]. Experimental studies found that the presence of the D allele is associated with higher levels of plasma ACE [8]. The higher levels of plasma ACE probably leads to a higher level of Ang-II and the disorder of the steroid hormone synthesis. To investigate whether the *ACE *genotype is associated with the abnormal levels of hormones in PCOS patients, we designed a case-control experiment including 346 PCOS patients and 236 non-PCOS individuals as controls.

## Methods

### Subjects

A total of 582 women were studied. 346 of them were patients with PCOS and 236 were non-PCOS women as controls. The controls were selected by excluding the diagnosis of PCOS according to the 2003 Rotterdam Criteria and exhibiting normal menstrual cycles (<32 days). All the individuals in the control group were without obesity, hirsutism (the F-G hirsutism score ≥ 7), acne, or overmuch sebum. All the women recruited for the study were from the Chinese Han ethnic group. The study was approved by Medical School of Nanjing University, and informed consent was obtained from every study participant.

### PCOS diagnostic criteria and hormone measurements

Patients with PCOS were diagnosed by the 2003 Rotterdam Criteria [16]. The Rotterdam Criteria requires at least two of the following indicators for diagnosis of PCOS: clinical or biochemical signs of hyperandrogenism (Table 1), oligomenorrhea or amenorrhea, and presence of polycystic ovarian (PCO) morphology on ultrasound (Table 1), with the exclusion of other causes of hyperandrogenism such as hyperprolactinemia, androgen-secreting tumors, Cushing's syndrome and nonclassic congenital adrenal hyperplasia. We calculated the body mass index (BMI = body weight in kilograms divided by square of height in meters) to assess obesity. The peripheral blood was obtained by a single venipuncture during the 3rd to the 5th day of the menstrual cycle for those who had menstruation and at any time for those who had amenorrhea. All peripheral blood samples were obtained between 8 AM and 9 AM after a 12-hour overnight fast. None of the study participants had been taking hormonal medications, including contraceptive pills, for the previous three months before the hormone measurement. Blood samples were immediately centrifuged, and then serum was separated and frozen at -80°C until assayed. Levels of total testosterone (T), follicle-stimulating hormone (FSH), luteinizing hormone (LH), total testosterone (T), and estradiol (E2) in the sera were measured by RIA (Beijing Northern Institute of Biological Technology of China and the CIS Company of France). The intra- and inter-assay coefficients of variation of all the assays were less than 10%.

**Table 1:** The criteria of clinical/biochemical hyperandrogenism and PCO

Clinical features	Hirsutism	There're pelages around anocheilon, lower mandible, areola mammae and the midline of inferior belly etc. Note:Although some patients have acnes, our gynecologists mainly rely on the hirsutism as the clinical features of hyperandrogenism.
Biochemical indices	Total testosterone (T) in the serum (nmol/L)	Normal: < 1.4; Hyperandrogenism: > 1.4(during the third to the fifth day of the menstrual cycle)

The criteria of PCO		1. The follicular phase ovary lacking follicles larger than 10 mm in diameter
		2. The presence of 12 or more follicles measuring 2–9 mm in diameter, or increased ovarian volume (>10 mL)

### Polymorphism genotype analysis

**Figure 1:**
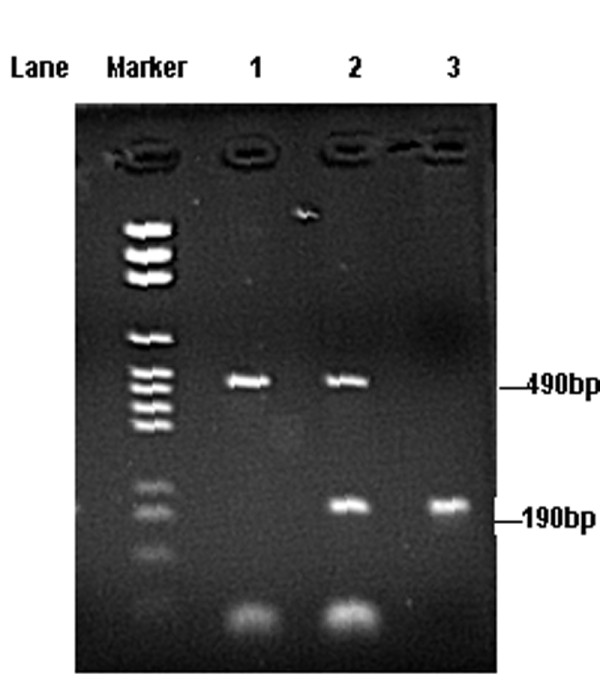
**PCR detection of the I/D polymorphism of the *ACE *gene (lanes 1–3)**. PCR conditions were as described by Brigitte Rigat et al [11]. 2% agarose gel electrophoresis, ethidium bromide staining and UV transillumination were performed. Lane Marker: DNA marker. Lane 1: Band of 490 bp indicating genotype II; Lane 2: Bands of 490 bp and 190 bp indicating genotype I/D; Lane 3: Band of 190 bp indicating genotype DD.

Genomic DNA was extracted from venous blood samples using Chelex^®^-100 as a medium (Promega, Madison, WI, USA). For each subject, the presence (allele I) or absence (allele D) of the 287-bp Alu repeat in intron 16 of the *ACE *gene was determined by measuring the size of DNA fragments after polymerase chain reaction (PCR) amplification. The PCR conditions were as described by Brigitte Rigat et al. [17], using a primer pair of 5'-CTG GAG ACC ACT CCC ATC CTT TCT-3' (sense oligo) and 5'-GAT GTG GCC ATC ACA TTC GTC AGA T-3' (anti-sense oligo). PCR amplification was carried out in a total volume of 25 μL containing 50 ng template DNA, 10 pmols of each primer, 2.5 μL STR (short tandem repeat) 10×buffer (STR 10×buffer, Promega, Madison, WI, USA) and 1.5 U of Taq DNA polymerase (Promega, Madison, WI, USA). The PCR was performed in a PTC-100 (MJ Research™, Incorporated) thermocycler as follows: An initial denaturation step of 2.5 minutes at 94°C, then 30 cycles consisting of 30 seconds of denaturation at 94°C, 105 seconds of annealing at 60°C, and 1.5 minutes of extension at 72°C. The PCR products were separated on a 2% agarose gel, and DNA was visualized by ethidium bromide staining (Figure 1). The expected PCR product was a 190-bp fragment in the presence of the deletion (D) allele and a 490-bp fragment in the presence of the insertion (I) allele. Thus, each DNA sample revealed one of three possible patterns after electrophoresis: a 490-bp band (genotype II), a 190-bp band (genotype DD), or both 490-and 190-bp bands (genotype ID).

### Statistical analysis

The frequencies of *ACE *alleles and genotypes among the patients and controls were obtained by direct enumeration based on the PCR results, and the deviation from Hardy-Weinberg equilibrium was evaluated using the χ2-test. Genotypic distributions between the patients and controls were compared by χ2-test of the 2 × 3 tables. The analysis was performed using the SAS system software (SAS Institute Inc., Cary, USA). The results of serum hormone levels were reported as MEANS ± SD. The regression and correlation tests were used to analyze the association of age or BMI and the concentrations of the hormone. Differences in serum hormone levels among different genotypic individuals were assessed by using one way analysis of variance (ANOVA). Tukey-test was used for further analysis of the differences among the three genotypes, which was performed using the SAS system software. P < 0.05 was considered statistically different.

## Results

The overall characteristics of the PCOS patients and the controls were presented in Table 2. There were no correlations between the age or BMI and the concentrations of the hormone. The frequencies of D allele were 51.45% and 55.3% in the patients (n = 346) and the controls (n = 236), respectively. In all the 582 study subjects, the *ACE *genotypic distributions were 28.32% (DD), 46.24% (ID), 25.43 (II) in the patients, and 32.63% (DD), 45.34% (ID), 22.03% (II) in the controls, respectively. In both the patient and control groups the genotypic distributions were in agreement with Hardy-Weinberg equilibrium (χ2 = 1.914 and 1.623, respectively, *P *> 0.05). No significant difference was observed in the frequency of the genotypic distributions between the patients with PCOS and the non-PCOS controls (*P *= 0.458) (Table 3).

**Table 2:** The overall characteristics of PCOS patients and controls

_	N	Age (years)	AAM (years)	BMI (kg/m^2^)	FSHIU/L	LHIU/L	LH/FSH	T (nMol/L)	E2 (pMol/L)
PCOS	346	26.49 ± 4.0*	14.57 ± 1.62	22.80 ± 3.79*	8.14 ± 5.91* (N = 325)	20.39 ± 20.3* (N = 325)	2.63 ± 1.33* (N = 325)	4.04 ± 4.12* (N = 325)	226.92 ± 117.04* (N = 325)
Control	236	31.64 ± 4.35	14.75 ± 2.66	21.32 ± 2.32	6.97 ± 2.16 (N = 219)	4.55 ± 2.16 (N = 219)	0.67 ± 0.33 (N = 219)	1.6 ± 3.82 (N = 219)	171.64 ± 140.68 (N = 219)

Note: *P < 0.05 vs control. We failed to assay the hormone levels in several women with PCOS and controls.

**Table 3:** Anthropometric characteristics and serum hormone concentrations in women with different genotypes

*ACE *genetype	PCOS				CONTROL			
				
	DD	ID	II	*P*	DD	ID	II	*P*
N	98	160	88	_	77	107	52	0.458^a^
Frequency (%)	28.32	46.24	25.43		32.63	45.34	22.03	
Age (years)	26.63 ± 3.86	26.31 ± 4.16	26.93 ± 3.69	0.75	32.62 ± 3.43	31.63 ± 4.45	31.15 ± 4.66	0.38
AAM (years)	14.43 ± 1.77	14.66 ± 1.61	14.44 ± 1.45	0.67	15.23 ± 3.73	14.59 ± 1.52	14.63 ± 2.76	0.58
BMI (kg/m^2^)	22.12 ± 3.23	23.15 ± 4.02	22.55 ± 3.66	0.33	20.75 ± 2.06	21.49 ± 2.46	21.48 ± 2.33	0.37
FSH (IU/L)	8.22 ± 5.00 (N = 92)	7.91 ± 4.5 (N = 151)	8.83 ± 10.25 (N = 83)	0.77	6.7 ± 2.87 (N = 72)	7.52 ± 2.3 (N = 99)	6.67 ± 1.49 (N = 48)	0.13
LH (IU/L)	20.94 ± 21.53 (N = 92)	21.98 ± 22 (N = 151)	13.96 ± 7.33 (N = 83)	0.19	4.68 ± 2.57 (N = 72)	4.77 ± 1.94 (N = 99)	4.31 ± 2.12 (N = 48)	0.55
LH/FSH	2.75 ± 1.6 (N = 92)	2.78 ± 1.26 (N = 151)	1.93 ± 0.88 (N = 83)	0.01	0.73 ± 0.34 (N = 72)	0.68 ± 0.37 (N = 99)	0.65 ± 0.3 (N = 48)	0.66
T* (nMol/L)	2.90 ± 1.11 (N = 92)	3.01 ± 1.32 (N = 151)	2.11 ± 0.9 (N = 83)	0.0045	1.62 ± 1.01 (N = 72)	1.03 ± 0.73 (N = 99)	1.11 ± 0.53 (N = 48)	0.0052
E_2 _(pMol/L)	259.7 ± 128.72 (N = 92)	223.49 ± 109.96 (N = 151)	190.46 ± 114.8 (N = 83)	0.053	191.52 ± 152.71 (N = 72)	189.84 ± 176.82 (N = 99)	147.35 ± 93.67 (N = 48)	0.25

Note: ^a:^*P *= 0.458, genotypic distributions in women with PCOS vs those in the controls.

P* = 0.0001 (the total T values of women with PCOS vs those of controls) We failed to assay the hormone levels in several women with PCOS and controls.

### Data analysis

By ANOVA analysis, we observed significant differences in the concentrations of testosterone among three genotypes in both the patients and the controls (*P *= 0.0045, *P *= 0.0052, respectively) (Table 3). Further analysis (Tukey) showed statistical differences (P < 0.05) between these groups with distinct genotypes: DD versus II and DI versus II in the PCOS patients as well as DD versus DI and DD versus II in the controls. In both patients and controls, the concentration of testosterone in the DD genotype was higher than that in the II genotype. Significant differences were also found in the ratio of LH/FSH among the three genotypes in the patients (*P *= 0.01). In contrast, no statistical differences in the concentration of estradiol (E2) were observed among the three genotypes in both patients and the controls (Table 3). We found no statistical differences of the BMI, AAM and other serum hormone concentrations among different genotypes in patients and controls.

## Discussion

In this case-control study the D allele of the *ACE *gene had no influence on the occurrence of PCOS. No significant differences were observed in the distribution of the genotypes between the patients and the controls. Regardless of the criterion used for assessing patients of PCOS (oligomenorrhea or amenorrhea; presence of polycystic ovarian (PCO) morphology on ultrasound, and clinical or laboratory evidence of hyperandrogenism), no differences were observed among the three genotypes. These results suggested that the D allele of the *ACE *gene was not a major etiological factor for PCOS. Our current finding is contrary to the observation reported by Yunxia Cao et al [18,19] in a smaller sample, which suggests that the D allele is associated with the enhanced RAS and the formation of polycystic ovary and hyperandrogen. Several possibilities may account for this discrepancy: first, the sample size in Cao's study was smaller, which may limit its representation of the colonia; second, the method chosen by Cao et al and us to detect the polymorphism may cause a misclassification of approximately 4 to 5% of the ID heterozygotes to the DD homozygotes [20]. We tried to correct this error. An additional PCR analysis is, therefore, needed for the confirmation of the DD genotypes obtained in the first standard PCR, including a new sense primer that is insertion-specific [20].

Although there were no significant differences in the distributions of the genotypes between the patients and the controls, we observed significant differences in the concentrations of testosterone among the three genotypes both in the patients and the controls (Table 3). By further analysis (Tukey-test), we found that, both in the patients and controls, the concentrations of testosterone in the DD genotype were higher than those in the II genotype (*P *< 0.05 respectively). The DD genotype was associated with a higher level of ACE than either the ID or II genotype [8], which implicates that the increased lever of ACE may be associated with the higher concentration of testosterone. It also indicates that the I/D polymorphisms of the *ACE *gene may directly or indirectly contribute to the expression of the testosterone in the ovaries. There can be a number of explanations for the phenomenon: firstly, the ACE is associated with the angiogenesis of ovarian endothelium in vitro [3]. Although there's no report about ACE promoting the angiogenesis of ovarian endothelium in vivo in human, the ovary has higher blood supply than the other organs in the body [21,22] and the majority of steroidogenic cells are in contact with at least one capillary [23]. If the higher level of ACE can stimulate the angiogenesis of ovarian endothelium in vivo and increase the blood supply, it may increase the supply of cholesterin which is the precursor of the steroid hormone. It is necessary to conduct further analysis to clarify the point. Secondly, the product of the ACE- Ang II is shown to inhibit LH-stimulated progesterone secretion from primary cultures of bovine corpora lutea cells [24] and from the midluteal phase bovine corpora lutea in an in vitro microdialysis system [25,26]. The reduced secretion of progesterone will stimulate the secretion of LH through negative feedback, which can explain the differential LH/FSH ratios in different genotypes of the patients. In addition, a higher level of testosterone can also increase the transformation of estrogen, which will further modulate the release of LH and FSH through either positive or negative feedback.

An interesting finding was that the total T concentrations between the I/D and D/D genotypes in the controls were different, whereas in the PCOS patients such concentrations between the I/D and I/I genotypes were different. One interpretation of this finding is that the I/D is a susceptible genotype in the population of hyperandrogenism. In the controls, the I/D and I/I genotypes demonstrate no differences in the concentrations of T. However, according to the prior studies, these two genotypes have different concentrations of ACE. Two possibilities may account for this observation: 1) the concentration of T is not associated with ACE; 2) ACE is not the key factor that influences the concentration of T. However, in the PCOS patients the total T concentrations between the I/D and I/I genotypes were different, which cannot be explained if ACE is not associated with the concentration of T. The most probable reason may be that the *ACE *I/D polymorphisms is only one of the co-factors that affects the etiopathogenisis of PCOS by interacting with other factors. The I/D genotype is more susceptible to hyperandrogenism.

## Conclusion

The I/D polymorphisms in the *ACE *gene was not associated with the pathogenesis of PCOS. However, the polymorphisms were associated with the steroidogenesis in the ovary. This suggests that although the I/D polymorphisms in the *ACE *gene were not the key etiological factor, it may be associated with the aggravated clinical manifestations of PCOS.

## Abbreviations

I/D Polymorphism: Insertion/Deletion Polymorphism; ACE: Angiotensin-converting Enzyme; RAS: the renin-angiotensin system; PCOS: Polycystic Ovary Syndrome; LH: luteinizing hormone; FSH: follicle-stimulating hormone; E2: estradiol; BMI: body mass index (body weight in kilograms divided by square of height in meters); AAM: age at menarche; T: testosterone; P: progesterone; RIA: radio-immunity assay; PCR: polymerase chain reaction; PCO: polycystic ovarian; Ang II: Angiotensin II.

## Competing interests

The authors declare that they have no competing interests.

## Authors' contributions

JS, HF, FL and CY carried out DNA extraction and the molecular genetics experiments. JS, LY, YW performed the statistical analysis and drafted the manuscript. YC, XW, HF and SH participated in sample collection. LY participated in the designing of the study and helped to carry out the molecular genetics experiments. YW and YC conceived and directed the study, and helped to draft the manuscript. All authors read and approved the final version of the manuscript.

## Pre-publication history

The pre-publication history for this paper can be accessed here:


